# Supplementary material for: Comparing pedestrian safety between electric and internal combustion engine vehicles
Source: Nat Commun. 2025 Dec 9;16:10824. doi: 10.1038/s41467-025-66463-8 (PMC12690075; doi:10.1038/s41467-025-66463-8)
Supplement: Supplementary file 4 — Description of Additional Supplementary Files [file 41467_2025_66463_MOESM4_ESM.docx]

**Description of Additional Supplementary Files**

**Comparing pedestrian safety between electric and**

**internal combustion engine vehicles**

Zia Wadud

Institute for Transport Studies, and School of Chemical and Process Engineering

University of Leeds, Leeds, LS2 9JT, UK

[Z.Wadud@leeds.ac.uk](mailto:Z.Wadud@leeds.ac.uk)

Supplementary Data 1 - Injury Severity Model Results. It presents the complete estimation results for binary logistic model (selected results of which is presented in Table 2 in main text). The file also contains the estimation results of an alternative binary probit model for pedestrian injury severity. Both models point to similar effect directions and statistical significance. The p-values and corresponding statistical significance are determined after applying Benjamini- Hocheberg’s false discovery rate correction in both models. Probit model coefficients are usually smaller than logit model coefficients.

Supplementary Data 2 - Summary Statistics. It presents the summary statistics of the explanatory variables for the injury severity model above (selected results of which is presented in Table 2 in main text). Since the variables are all categorical, only share of different levels describing a variable is presented (instead of mean and standard deviation).
